# Hepatitis B virus X protein induces E6-associated protein-mediated proteasomal degradation of p53 phosphorylated at Ser-15

**DOI:** 10.1099/jgv.0.002291

**Published:** 2026-06-30

**Authors:** Jiwoo Han, Yerin Kwon, Ji-Min Park, Kyung Lib Jang

**Affiliations:** 1 Department of Integrated Biological Science, The Graduate School, Pusan National University, Busan 46241, Republic of Korea; 2 Department of Microbiology, College of Natural Science, Pusan National University, Busan 46241, Republic of Korea; 3 Microbiological Resource Research +Institute, Pusan National University, Busan 46241, Republic of Korea

**Keywords:** E6-associated protein, HBx, hepatitis B virus, p53, proteasomal degradation, ubiquitination

## Abstract

The hepatitis B virus (HBV) X protein (HBx) activates the ataxia telangiectasia mutated (ATM)-checkpoint kinase 2 (Chk2) pathway, leading to phosphorylation of p53 at multiple sites including Ser-15. This phosphorylation largely contributes to p53 accumulation by preventing its degradation via MDM2. This study further shows that HBx facilitates E6-associated protein (E6AP)-mediated proteasomal degradation of p53 during HBV replication. In the presence of HBx, E6AP expression induced p53 ubiquitination, reduced its stability and decreased p53 levels, whereas E6AP knockdown elevated p53 levels. The critical role of E3 ligase activity in E6AP was confirmed using the E6AP (C833A) mutant, the homologous to the E6-AP Carboxyl Terminus (HECT)-type ubiquitin E3 ligase inhibitor Heclin and the proteasome inhibitor MG132. E6AP-mediated p53 degradation was severely impaired in the presence of the ATM inhibitor KU-55933, indicating that HBx-induced p53 phosphorylation plays a critical role in this process. Additionally, E6AP could target p53 phosphorylated by DNA-damaging agents like etoposide in the absence of HBx. HBx also strengthened the interaction between phosphorylated p53 and E6AP, suggesting an additional mechanism that enhances E6AP-mediated ubiquitination of phosphorylated p53. Ser-15 phosphorylation was pivotal, as E6AP could degrade p53 S15D (phosphomimetic mutant) but failed to act on p53 S15A (a non-phosphorylatable mutant). These findings suggest that HBx-induced p53 phosphorylation enhances E6AP-mediated degradation while concurrently inhibiting MDM2-mediated pathways, thereby fine-tuning p53 levels to support cell survival, viral replication and potentially carcinogenesis during HBV infection in human hepatocytes.

## Introduction

Hepatitis B virus (HBV) is a major human hepatotropic pathogen that causes both acute and chronic hepatitis, which can lead to severe liver complications such as cirrhosis, liver failure and hepatocellular carcinoma (HCC) [1]. As a representative member of the family Hepadnaviridae, HBV replicates its partially double-stranded circular DNA genome of ~3.2 kbp by reverse transcription of a pregenomic RNA [2,3]. Among the four ORFs – S, C, P and X – in the HBV genome, the smallest, X, encodes a multifunctional regulatory protein of 154 amino acids called the HBV X protein (HBx). HBx has been implicated in the development of HBV-associated HCC because of its roles in modulating signalling pathways, activating transcription of cellular genes and disrupting cell proliferation, apoptosis, DNA repair and immune responses [4,6]. Although knowledge of HBx’s functions in HBV oncogenesis has grown, the exact mechanism by which HBx exerts its oncogenic effects remains unclear.

p53, often called ‘the guardian of the genome’, is a key tumour suppressor protein that prevents cancer development [7]. As a transcription factor, p53 responds to cellular stresses – including DNA damage, oncogene activation and hypoxia – by activating pathways that lead to cell cycle arrest, apoptosis, senescence and DNA repair [8,9]. This broad range of responses helps p53 maintain genomic stability and prevents the spread of damaged cells, underscoring its essential role in cancer prevention. Normally, p53 levels remain low due to rapid degradation, primarily mediated by the E3 ubiquitin ligase MDM2 [10,11]. When cells experience stress, the ataxia telangiectasia mutated (ATM)-checkpoint kinase 2 (Chk2) pathway is activated, leading to the phosphorylation of p53 and MDM2. It is well known that this phosphorylation disrupts the MDM2–p53 complex and stabilizes p53 by preventing MDM2-mediated ubiquitination [10,13], but it remains unclear whether phosphorylated p53 is still degraded. This issue is especially relevant when maintaining p53 levels within a specific range is necessary, such as in cells exposed to HBV infection.

The interaction between p53 and HBx appears essential to their respective roles: p53 as a host tumour suppressor and HBx as a viral oncoprotein. The antiviral function of p53 against HBV has been confirmed by studies showing that p53 expression markedly decreases viral replication in cell culture systems [14,15]. Moreover, HBV replicates more efficiently in cells with p53 knockdown or mutation [15,16]. Specifically, p53 reduces HBx levels by transcriptionally activating Siah-1, the first known E3 ligase targeting HBx [16,17]. To counter p53’s role in anti-HBV defence, HBx may have evolved a mechanism to inactivate p53, similar to mechanisms used by the SV40 large T antigen and human adenovirus E1B-58K [18]. In fact, HBx binds to p53 and inhibits several key p53 functions, including nuclear entry, DNA-sequence-specific binding and transcriptional activation [19,21]. Interestingly, HBV infection – mainly through HBx – also triggers cellular stress signals, particularly via ER and mitochondrial dysfunction, leading to reactive oxygen species production [22,23], which in turn elevates p53 levels by activating the ATM-Chk2 pathway [16,24]. Consequently, HBx needs to evolve mechanisms to lower p53 levels, thereby supporting cell survival, viral replication and potentially oncogenesis during HBV infection in human liver cells. However, it remains unknown whether HBx can downregulate p53 levels by inducing its degradation via the proteasome, as demonstrated by the E6 of human papillomaviruses (HPVs) [25,26] and the E1B-55K of human adenoviruses [27].

E6-associated protein (E6AP) was initially identified as an E3 ligase that binds p53 and the E6 proteins of HPV-16 and HPV-18, leading to p53 degradation [25,26]. E6AP has also been shown to interact with HBx as a second E3 ligase, resulting in HBx ubiquitination and degradation [15,17]. Since p53 is also known to interact with HBx [19,20], it is possible that HBx, E6AP and p53 form a trimeric complex that induces p53 degradation, similar to the HPV E6–E6AP–p53 interaction [28,29]. Moreover, recent findings suggest that E6AP can target p53 for ubiquitination when p53 is phosphorylated in response to genotoxic stress [30]. Since HBx is known to induce p53 phosphorylation through activation of the ATM-Chk2 pathway [16,24], it is plausible that HBx facilitates E6AP-mediated p53 degradation by phosphorylating p53. This study aims to explore several key questions related to these hypotheses. First, we investigated whether HBx induces E6AP-mediated ubiquitination and proteasomal degradation of p53. Second, we compared the roles of MDM2 and E6AP in p53 degradation, both in the presence and absence of HBx. Third, we examined how HBx facilitates E6AP-mediated p53 degradation, with a specific focus on its ability to induce p53 phosphorylation. The potential for HBx to enhance the interaction between E6AP and p53 was also considered. Fourth, we assessed whether p53 phosphorylation is essential for HBx-mediated ubiquitination of p53 by E6AP. Finally, we attempted to identify the critical phosphorylation site(s) in p53 that make it susceptible to E6AP-mediated ubiquitination. Understanding the fate of phosphorylated p53, especially under stress conditions like HBV infection, is crucial for elucidating how p53 levels are tightly regulated to balance cell survival and viral replication.

## Methods

### Plasmids

The plasmid pCMV-3×HA1-HBX3 encodes full-length HBx (genotype D) downstream of three copies of the influenza virus haemagglutinin (HA) [31]. The 1.2-mer WT HBV replicon, containing 1.2 units of the HBV genome (genotype D), and its HBx-null counterpart [32] were kindly provided by W.S. Ryu (Yonsei University, Seoul, Republic of Korea). Plasmids pCMV-3×HA-hE6AP (Cat. No. 37601), which encodes HA-tagged E6AP (amino acids 262–853); pcDNA3-MDM2 WT (Cat. No. 16233), which encodes the WT human MDM2 (amino acids 1–491); and pCH110 (Cat. No. 27-4508-01), which encodes the *Escherichia coli* β-galactosidase (β-gal), were obtained from Addgene (Watertown, MA, USA). The plasmid RC210241 (Cat. No. 003049), encoding the human Na^+^-taurocholate cotransporting polypeptide (NTCP), was obtained from OriGene (Rockville, MD, USA). The pCMV p53-WT and pHA-Ub were gifts from C.-W. Lee (Sungkyunkwan University, Suwon, Republic of Korea) and Y. Xiong (UNC-CH, Chapel Hill, NC, USA), respectively. Additionally, pcDNA3 p53 S15A (Cat. No. 69004) and pcDNA3 p53 S15D (Cat. No. 69005) were purchased from Addgene. Using PCR-directed mutagenesis, plasmids encoding p53 S20D, p53 S15A/S20D and p53 S15D/S20D were generated from pCMV p53-WT, pcDNA3 p53 S15A and pcDNA3 p53 S15D, respectively. Scrambled small hairpin RNA (shRNA; Cat. No. sc-42964) and E6AP shRNA (Cat. No. sc-43742) were obtained from Santa Cruz Biotechnology (Santa Cruz, CA, USA). For the mammalian two-hybrid assay, PCR fragments from pCMV-3×HA-hE6AP and pcDNA3-MDM2 WT encoding E6AP (amino acids 262–853) and MDM2 (amino acids 1–491) were cloned into the pSG424 plasmid [33], in-frame and downstream of Gal4 (amino acids 1–147), to generate G4-E6AP and G4-MDM2, respectively. Additionally, a PCR fragment encoding p53-WT (amino acids 1–393) from pCMV p53-WT was fused upstream of the VP16 activation domain (amino acids 423–490) in pCMV-VP16 [33] to generate pCMV p53-VP16. The reporter plasmid G5E1b-luc [34] contains five Gal4 DNA-binding sites upstream of a minimal E1b promoter in the pGL3 vector (Promega, Cat. No. E1751, Madison, WI, USA).

### Cell culture

Cells were cultured in Dulbecco’s modified Eagle medium (DMEM; WelGENE, Cat. No. LM001-05, Gyeongsan, Republic of Korea) supplemented with 10% FBS (Capricorn Scientific, Cat. No. FBS-22A, Ebsdorfergrund, Germany), 100 units ml^−1^ of penicillin G (Sigma-Aldrich, Cat. No. P3032, Saint Louis, MO, USA) and 100 µg ml^−1^ streptomycin (United States Biological, Cat. No. 21865, Salem, MA, USA) in a humidified 5% CO₂ atmosphere at 37 °C. The HCC cell lines, Hep3B (Cat. No. 88064) and HepG2 (Cat. No. 88065), were obtained from the Korean Cell Line Bank (Seoul, Republic of Korea). The HepG2-HBx stable cell line was established by transfecting HepG2 cells with pCMV-3×HA1 HBx, followed by selection with 500 µg ml^−1^ G418 sulphate (Sigma-Aldrich, Cat. No. A1720). Additionally, HepG2-NTCP and Hep3B-NTCP cell lines, which stably express the NTCP transporter, were constructed similarly. For transient expression experiments, 2×10⁵ cells in a six-well plate were transfected using the TurboFect transfection reagent (Thermo Fisher Scientific, Cat. No. R0532, Waltham, MA, USA). When necessary, cells were treated with KU-55933 (Abcam, Cat. No. ab120637, Cambridge, UK), MG132 (Millipore, Cat. No. 474790, Burlington, MA, USA), cycloheximide (CHX) (Sigma-Aldrich, Cat. No. C7698), etoposide (Sigma-Aldrich, Cat. No. E1383) or Heclin (Sigma-Aldrich, Cat. No. SML1396) under the indicated conditions.

### HBV cell culture system

HBV stocks were prepared as previously described [15,35]. Briefly, Hep3B-NTCP cells were transiently or stably transfected with the 1.2-mer HBV replicon plasmid as described above. The culture supernatant was collected to determine HBV titres, as previously described [36]. HBV infection experiments were performed in six-well plates at an m.o.i. of 50 for 4 days using an optimized HBV cell culture system [15,35, 37]. Briefly, 2×10⁵ cells were exposed to HBV at an m.o.i. of 1×10⁷ and incubated for 24 h in DMEM supplemented with 4% polyethylene glycol (PEG) 8000 (Sigma-Aldrich Cat No. D4463) and 2% DMSO (Sigma-Aldrich Cat No. D8418). After two washes with serum-free DMEM, cells were maintained in DMEM with 3% FBS, 4% PEG 8000 and 2% DMSO for an additional 3 days.

### Co-immunoprecipitation

Co-immunoprecipitation (Co-IP) assays were performed using a Classic Magnetic IP/Co-IP Kit (Thermo Fisher Scientific, Cat. No. 88804). Briefly, ~1×10^6^ cells per 100 mm dish were either transiently transfected with the indicated plasmids for 48 h or infected with HBV under the conditions described above. Whole-cell lysates (500 µg) were incubated with antibodies against total p53 (Cell Signalling Technology, Cat. No. 2527, Danvers, MA, USA), E6AP (Thermo Fisher Scientific, Cat. No. PA3-843), MDM2 (Santa Cruz Biotechnology, Cat. No. sc-965) and pSer-15 p53 (Cell Signalling Technology, Cat. No. 9284) for 12 h at 4 °C to form immune complexes. After washing, immune complexes were collected using Protein A/G magnetic beads (0.25 mg) by incubating for an additional hour with mixing. The beads were harvested using a magnetic stand (Pierce, Waltham, MA, USA), and the antigen/antibody complexes were then analysed by Western blotting with anti-HA or other relevant antibodies.

### Western blot analysis

Cell lysates were prepared in a buffer containing 50 mM Tris/HCl (pH 7.5), 150 mM NaCl, 0.1% SDS and 1% NP-40, supplemented with protease inhibitors. Protein concentration in each lysate was measured using a protein assay reagent (Bio-Rad, Cat. No. 5000006, Hercules, CA, USA). Proteins were separated by SDS-PAGE and transferred to nitrocellulose membranes (Cytiva, Cat. No. 10600004, Little Chalfont, UK). After blocking, membranes were incubated with primary antibodies against total p53 (Santa Cruz Biotechnology, Cat. No. sc-126, 1:1,000), pSer-15 p53 (Cell Signalling Technology, Cat. No. 9284, 1:1,000), pSer-20 p53 (Cell Signalling Technology, Cat. No. 9285, 1:1,000), HBx (Millipore, Cat. No. MAB8419, Burlington, MA, USA, 1:1,000), E6AP (Thermo Fisher Scientific, Cat. No. PA3-843, 1:2,000), MDM2 (Santa Cruz Biotechnology, Cat. No. sc-965, 1:1,000), pSer-1981 ATM (Abcam, Cat. No. ab5883, 1:1,000), total ATM (Abcam, Cat. No. ab2873, 1:1,000), pThr-68 Chk2 (Abcam, Cat. No. ab2661, 1:1,000), total Chk2 (Abcam, Cat. No. ab2662, 1:1,000), HA (Santa Cruz Biotechnology, Cat. No. sc-7392, 1:500) and γ-tubulin (Santa Cruz Biotechnology, Cat. No. sc-17787, 1:500). The membranes were incubated with horseradish peroxidase-conjugated secondary antibodies including anti-mouse IgG (Bio-Rad, Cat. No. BR170-6516, 1:3,000), anti-rabbit IgG (Bio-Rad, Cat. No. BR170-6515, 1:3,000) and anti-goat IgG (Thermo Fisher Scientific, Cat. No. 31400, 1:10,000) for 1 h. Protein bands were detected using an enhanced chemiluminescence kit (Advansta, Cat. No. K-12043-D20, San Jose, CA, USA) and imaged on a ChemiDoc XRS system (Bio-Rad). The target protein images were cropped from the original images to display the protein bands in the figure.

### Luciferase reporter assay

A total of ~1×10⁵ cells per well in 12-well plates were transfected with 0.2 µg of the reporter plasmid and the assigned plasmids under the indicated conditions. To control for variation in transfection efficiency, 0.1 µg of pCH110 was co-transfected as an internal control. Forty-eight hours after transfection, luciferase activity was measured using the Luciferase Reporter 1000 Assay System (Promega, Cat. No. E4550) and a microplate luminometer (LuBi, MicroDigital, Seongnam, Republic of Korea). Luciferase activity was normalized to β-gal activity measured in the corresponding cell extracts using a β-gal assay kit (Thermo Fisher Scientific, Cat. No. K1455-01).

### Statistical analysis

Data are presented as mean±sd and are derived from at least three independent experiments. Statistical analysis was performed using a two-tailed Student’s t-test in SigmaPlot (version 12.5). Significance was assessed using *P*-values, with *P*≤0.05 considered statistically significant.

## Results

### E6AP downregulates p53 levels in an HBx-dependent manner

**Fig. 1.**
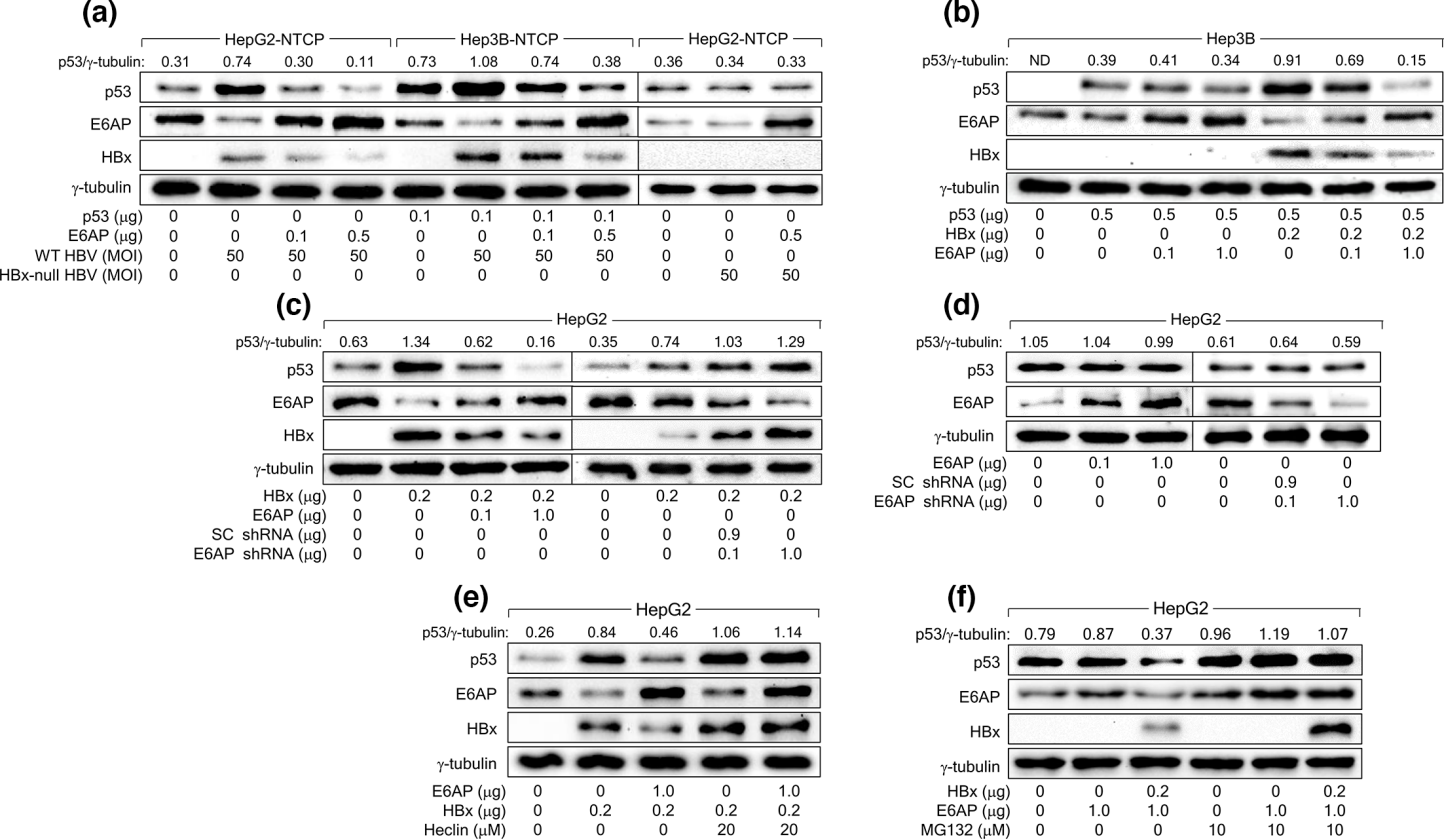
E6AP downregulates p53 levels in an HBx-dependent manner. (**a**) HepG2-NTCP and Hep3B-NTCP cells were transiently transfected with an empty vector, a p53 expression plasmid or an E6AP expression plasmid for 24 h, then infected with WT HBV or HBx-null HBV at the indicated m.o.i. as described in Methods. (b–f) HepG2 and Hep3B cells were transiently transfected with an empty vector or an HBx expression plasmid, together with the indicated plasmids, for 48 h, followed by Western blotting. (**e, f**) Cells were treated with Heclin for 12 h or with MG132 for 4 h before harvesting. p53 and γ-tubulin band intensities were quantified using ImageJ (NIH). Values indicate p53 levels relative to the loading control (γ-tubulin).

Initially, we investigated whether HBV infection affects E6AP’s ability to downregulate p53 in human hepatoma cells. For *in vitro* HBV infection, we used two human hepatoma cell lines, HepG2-NTCP and Hep3B-NTCP [15], both of which stably express the HBV entry receptor NTCP [38]. Infection with WT HBV increased endogenous p53 in HepG2-NTCP cells and exogenous p53 in Hep3B-NTCP cells (Fig. 1a). HBV infection also decreased E6AP levels in both cell lines. In contrast, in HepG2-NTCP cells infected with HBx-null HBV, neither an increase in p53 nor a decrease in E6AP was observed. The role of HBx was further supported by ectopic HBx expression, which alone was sufficient to elevate p53 levels and reduce E6AP levels in p53-expressing HepG2 and Hep3B cells (Fig. 1b, c). These results confirm our prior reports that HBx increases p53 levels while reducing E6AP levels during HBV infection [15,35].

Ectopic E6AP expression dose-dependently decreased both endogenous and exogenous p53 levels during WT HBV infection in HepG2-NTCP and Hep3B-NTCP cells, respectively (Fig. 1a). However, this effect was not observed in HepG2-NTCP cells infected with HBx-null HBV. Additionally, in the presence of HBx, ectopic E6AP expression lowered p53 levels, whereas E6AP knockdown increased p53 levels in HepG2 and Hep3B cells (Fig. 1b−d). Without HBx, neither E6AP expression nor knockdown significantly changed p53 levels in human hepatoma cells (Fig. 1b, d), supporting previous findings that E6AP alone cannot induce the proteasomal degradation of p53 [39,40]. Ectopic E6AP expression also decreased HBx levels, whereas E6AP knockdown raised HBx levels (Fig. 1a−c), consistent with earlier reports showing that E6AP decreases HBx levels in the presence of p53 [15]. These results indicate that E6AP lowers p53 levels in human hepatoma cells in an HBx-dependent manner.

### E6AP induces ubiquitin-dependent proteasomal degradation of p53 in an HBx-dependent manner

E6AP functions as an HECT-type E3 ubiquitin ligase [41] and is crucial for targeting p53 for proteasomal degradation. Treatment with the homologous to the E6-AP Carboxyl Terminus (HECT) E3 ubiquitin ligase inhibitor Heclin [42] not only increased p53 levels but also prevented E6AP-mediated degradation of p53 in the presence of HBx (Fig. 1e). Similarly, treatment with the universal proteasomal inhibitor MG132 blocked E6AP’s ability to decrease p53 levels in the presence of HBx (Fig. 1f). These results show that E6AP drives the proteasomal degradation of p53 in an HBx-dependent manner. Additionally, treatment with Heclin and MG132 increased HBx levels, consistent with previous reports indicating that HBx is a target of E6AP-mediated proteasomal degradation [15].

**Fig. 2.**
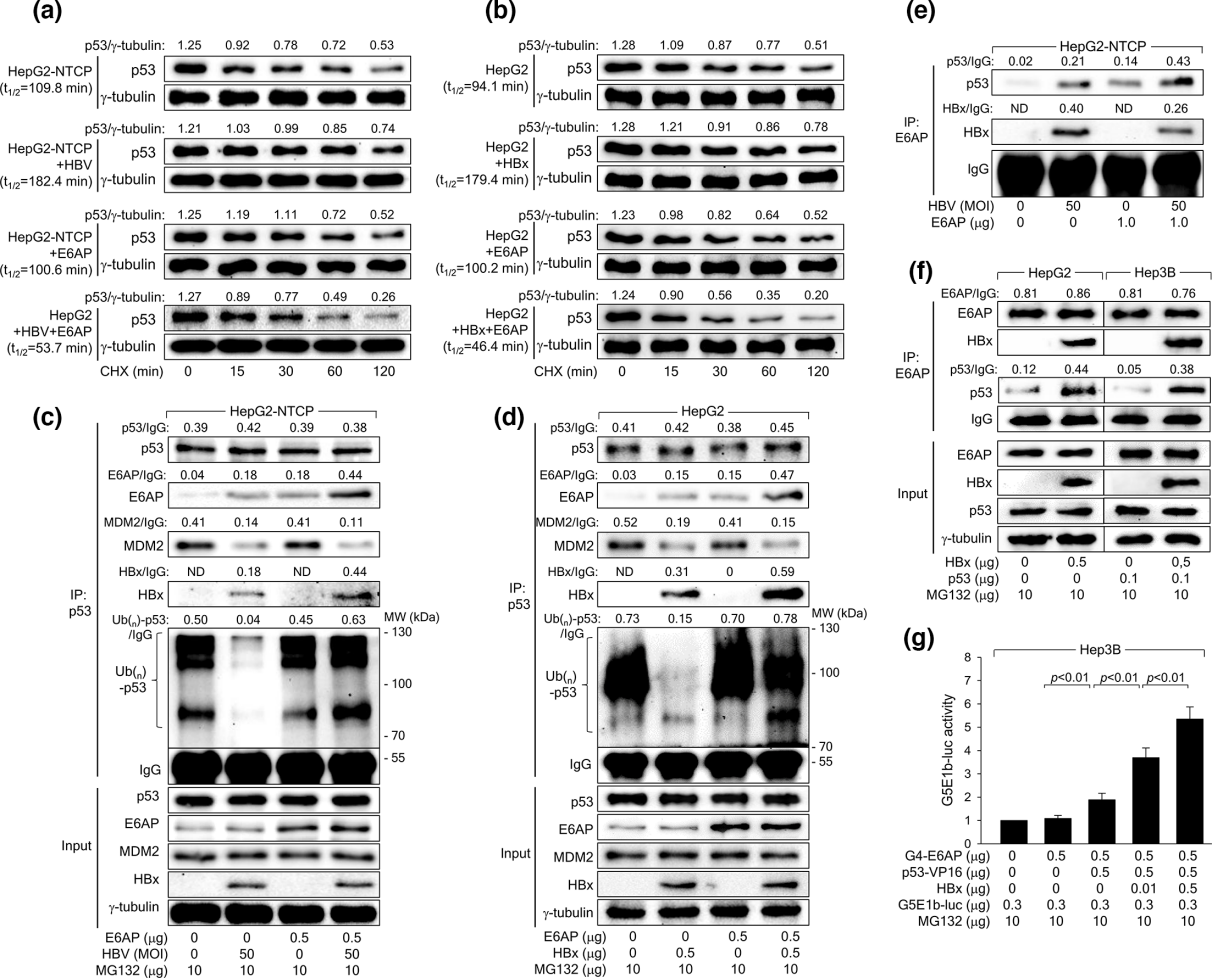
HBx stimulates E6AP-mediated proteasomal degradation of p53 during HBV replication. (**a, c, e**) HepG2-NTCP cells were either mock-infected or infected with WT HBV, as described in Fig. 1a. (**b, d, f**) HepG2 cells were transfected with an empty vector or an HBx expression plasmid, along with the indicated plasmids, for 48 h. (**a, b**) Cells were treated with 50 µM CHX for the indicated times before harvesting, followed by Western blotting. p53 and γ-tubulin bands were quantified to determine the half-life (t_1/2_) of p53. (c–f) Cell extracts were immunoprecipitated with either an anti-p53 antibody or an anti-E6AP antibody and subjected to Western blotting using anti-p53, anti-E6AP, anti-MDM2, anti-HBx and anti-HA antibodies to detect p53, E6AP, MDM2, HBx and HA-Ub-complexed p53, respectively. The input indicates the levels of the designated proteins in the cell lysates. (**g**) For mammalian two-hybrid assays, Hep3B cells were transfected with the Gal4 reporter (G5E1b-luc), pSG424-E6AP and pCMV p53-VP16, along with either an empty vector or an HBx expression plasmid, for 48 h, followed by a luciferase assay. Cells were treated with 10 µM MG132 for 4 h before harvesting. Luciferase activity from G5E1b-luc was normalized to the β-gal activity measured in the corresponding cell extract. The values show relative luciferase activity compared to the basal level. Results are presented as mean±sd from four independent experiments (*n*=4). nd, not detected.

We further investigated whether E6AP affects p53 protein stability in human hepatoma cells, with and without HBx. To do this, we treated HepG2-NTCP cells, whether or not infected with HBV, with CHX to inhibit protein synthesis and measured p53 and γ-tubulin levels. In HepG2-NTCP cells, p53 had a typical half-life (t_1/2_) of 109.8 min (Fig. 2a). HBV infection extended the t_1/2_ of p53 to 182.4 min. Overexpressing HBx alone was sufficient to produce this effect in HepG2 cells (Fig. 2b), confirming previous reports that HBx increases p53 stability during HBV replication [15,16, 24]. While overexpression of E6AP alone did not significantly change the t_1/2_ of p53 in HepG2-NTCP cells, it reduced the t_1/2_ to 53.7 min in HBV-infected cells (Fig. 2a). The effect of E6AP on p53 stability was consistently observed in an HBx overexpression system (Fig. 2b), indicating that E6AP decreases p53 stability in an HBx-dependent manner.

Next, we examined whether HBV infection influences E6AP-mediated p53 ubiquitination in HepG2-NTCP cells. Proteasomal inhibition with MG132 eliminated the effect of HBx on p53 levels (Fig. 2c, d), confirming that HBx mainly increases p53 levels by preventing its proteasomal degradation [15,16, 24]. In the presence of MG132, HBx no longer reduced MDM2 and E6AP levels, and E6AP could not lower HBx levels during HBV infection, indicating that these effects also depend on the proteasome. Co-IP experiments with an anti-p53 antibody revealed that MDM2, rather than E6AP, preferentially interacts with p53 to cause its ubiquitination in uninfected HepG2-NTCP cells, as shown by polyubiquitinated p53 bands (Fig. 2c, lane 1). Although overexpressing E6AP slightly increased its binding to p53 without affecting the MDM2–p53 interaction, it did not significantly change p53 ubiquitination in uninfected cells (Fig. 2c, lane 3). These findings suggest that MDM2, not E6AP, primarily mediates p53 ubiquitination in uninfected cells. HBV infection significantly reduced the interaction between MDM2 and p53, resulting in a sharp decrease in polyubiquitinated p53 levels (Fig. 2c, lane 2). HBV infection clearly enhanced E6AP binding to p53, but the effect was insufficient to affect p53 ubiquitination, likely due to low E6AP levels in infected cells. Indeed, overexpression of E6AP markedly increased its binding to p53, resulting in a notable rise in p53 ubiquitination in HBV-infected cells (Fig. 2c, lane 4). These data indicate that E6AP plays a key role in p53 ubiquitination during HBV infection. The roles of HBx, MDM2 and E6AP in p53 ubiquitination were consistently observed in an HBx-overexpression system (Fig. 2d). Additionally, both co-IP with an anti-E6AP antibody and mammalian two-hybrid assays provided further evidence that HBx promotes the binding of E6AP to p53 (Fig. 2e−g). Overall, we conclude that E6AP facilitates p53 ubiquitination in an HBx-dependent manner, whereas MDM2 mediates this process in the absence of HBx.

### E6AP induces ubiquitin-dependent proteasomal degradation of phosphorylated p53 in the presence of HBx

**Fig. 3.**
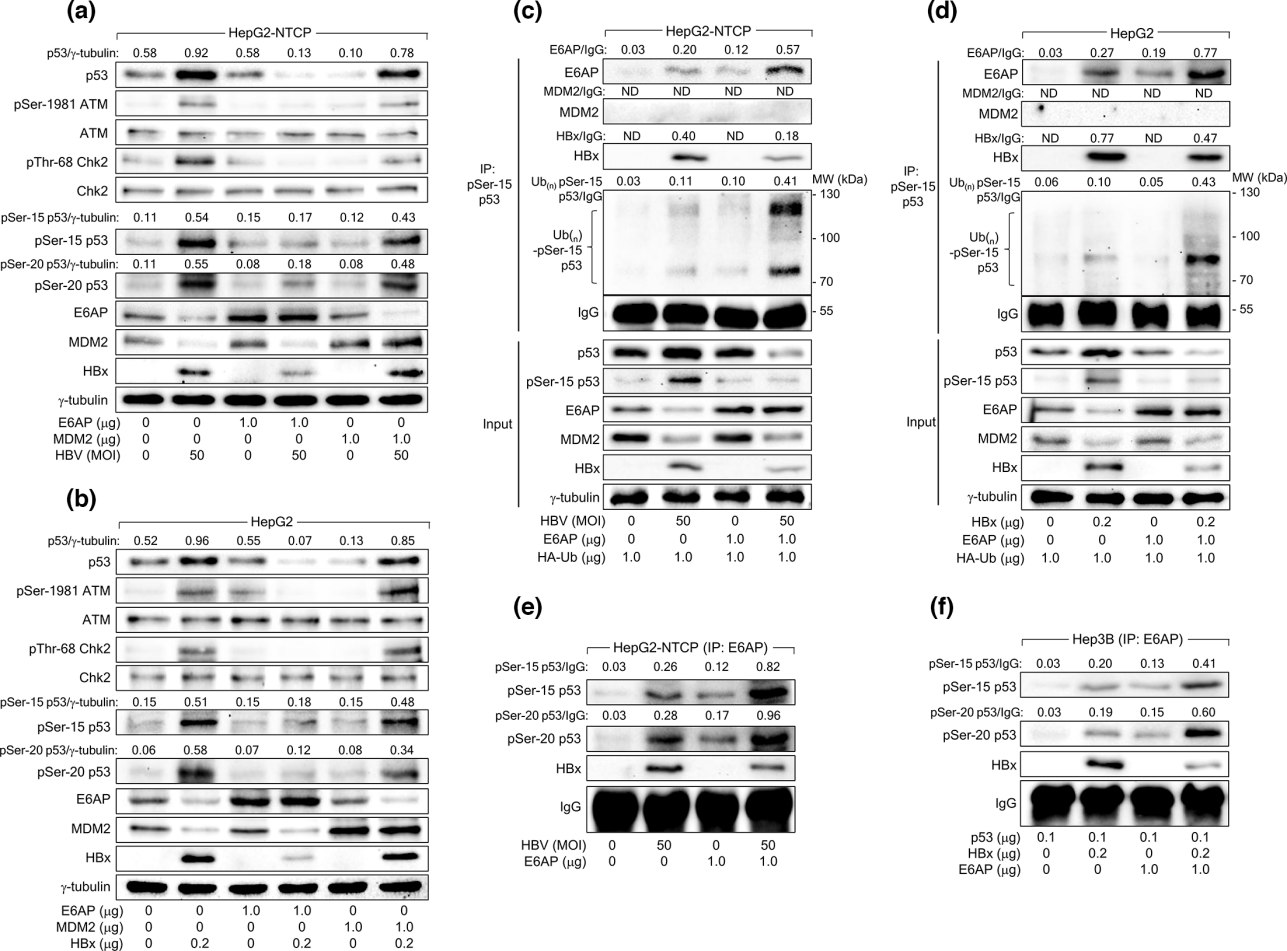
E6AP induces ubiquitin-dependent proteasomal degradation of phosphorylated p53 in the presence of HBx. (**a, c, e**) HepG2-NTCP cells were either mock-infected or infected with WT HBV, as described in Fig. 1a. (**b, d, f**) HepG2 cells were transfected with an empty vector or an HBx expression plasmid, along with the indicated plasmids, for 48 h. (**a, b**) Protein levels were measured by Western blot analysis. (**c, d**) Phosphorylated p53 at Ser-15 (pSer-15 p53) in cell lysates was immunoprecipitated with an anti-pSer-15 p53 antibody and subjected to Western blotting with anti-E6AP, anti-MDM2, anti-HBx and anti-HA antibodies to detect E6AP, MDM2, HBx and HA-Ub-complexed pSer-15 p53, respectively. The input indicates the levels of the designated proteins in the cell lysates. (**e, f**) Cell extracts were immunoprecipitated with an anti-E6AP antibody and subjected to Western blotting with anti-pSer-15 p53, anti-pSer-20 p53 and anti-HBx antibodies to detect pSer-15 p53, pSer-20 p53 and HBx, respectively.

Consistent with previous reports showing that HBx upregulates p53 levels by activating the ATM-Chk2 pathway [16,24], both HBV infection in HepG2-NTCP and HBx overexpression in HepG2 cells induced ATM phosphorylation at Ser-1981 without altering its total protein levels (Fig. 3a, b). Activated ATM then triggered phosphorylation of p53 at Ser-15 and of Chk2 at Thr-68, without affecting their total protein levels, leading to phosphorylation of p53 at Ser-20. These phosphorylation events increased p53 levels by stabilizing the protein in HepG2-NTCP and HepG2 cells, as previously reported [15,16, 24].

We investigated whether phosphorylation of p53 influences E6AP-mediated p53 degradation in human hepatoma cells. Both HBV infection and HBx expression decreased E6AP and MDM2 levels (Fig. 3a, b, lane 2), consistent with earlier reports [15,35, 43]. Ectopic E6AP expression markedly reduced total and phosphorylated p53 levels when HBx was present but had no effect in its absence (Fig. 3a, b, lanes 3 and 4). Additionally, E6AP lowered phosphorylated ATM and Chk2 levels only in the presence of HBx, likely because the p53-mediated feedback amplification of the ATM-Chk2 pathway [44] was compromised. In contrast, ectopic MDM2 expression decreased total p53 levels only in the absence of HBx, with minimal impact on phosphorylated ATM, Chk2 and p53 (Fig. 3a, b, lanes 5 and 6), as previously shown [45]. These results suggest that E6AP, unlike MDM2, facilitates the degradation of phosphorylated p53 in an HBx-dependent manner.

To confirm that E6AP ubiquitinates phosphorylated p53 in the presence of HBx, we performed co-IP experiments using antibodies against p53 phosphorylated at Ser-15 (pSer-15 p53) (Fig. 3c, d) and against E6AP (Fig. 3e, f). In the absence of HBx, E6AP showed minimal interaction with pSer-15 p53 and/or pSer-20 p53, likely reflecting low background p53 phosphorylation levels (Fig. 3c−f). HBx from both infection and overexpression systems enhanced the interaction between E6AP and phosphorylated p53, primarily by inducing p53 phosphorylation and increasing levels of polyubiquitinated pSer-15 p53. HBx also interacted with pSer-15 p53 and E6AP, indicating the formation of a trimeric complex. Ectopic E6AP expression slightly increased its binding to pSer-15 p53 and promoted ubiquitination of phosphorylated p53 even in the absence of HBx; however, this effect was more pronounced when HBx induced p53 phosphorylation at multiple sites, including Ser-15 (Fig. 3c−f). Unlike E6AP, MDM2 did not bind pSer-15 p53, regardless of HBx presence, in either the HBV infection or HBx overexpression systems (Fig. 3c, d). These results lead us to conclude that, when HBx is present, E6AP, not MDM2, primarily ubiquitinates phosphorylated p53.

### p53 phosphorylation is required for E6AP-mediated ubiquitination of p53 in the presence of HBx

**Fig. 4.**
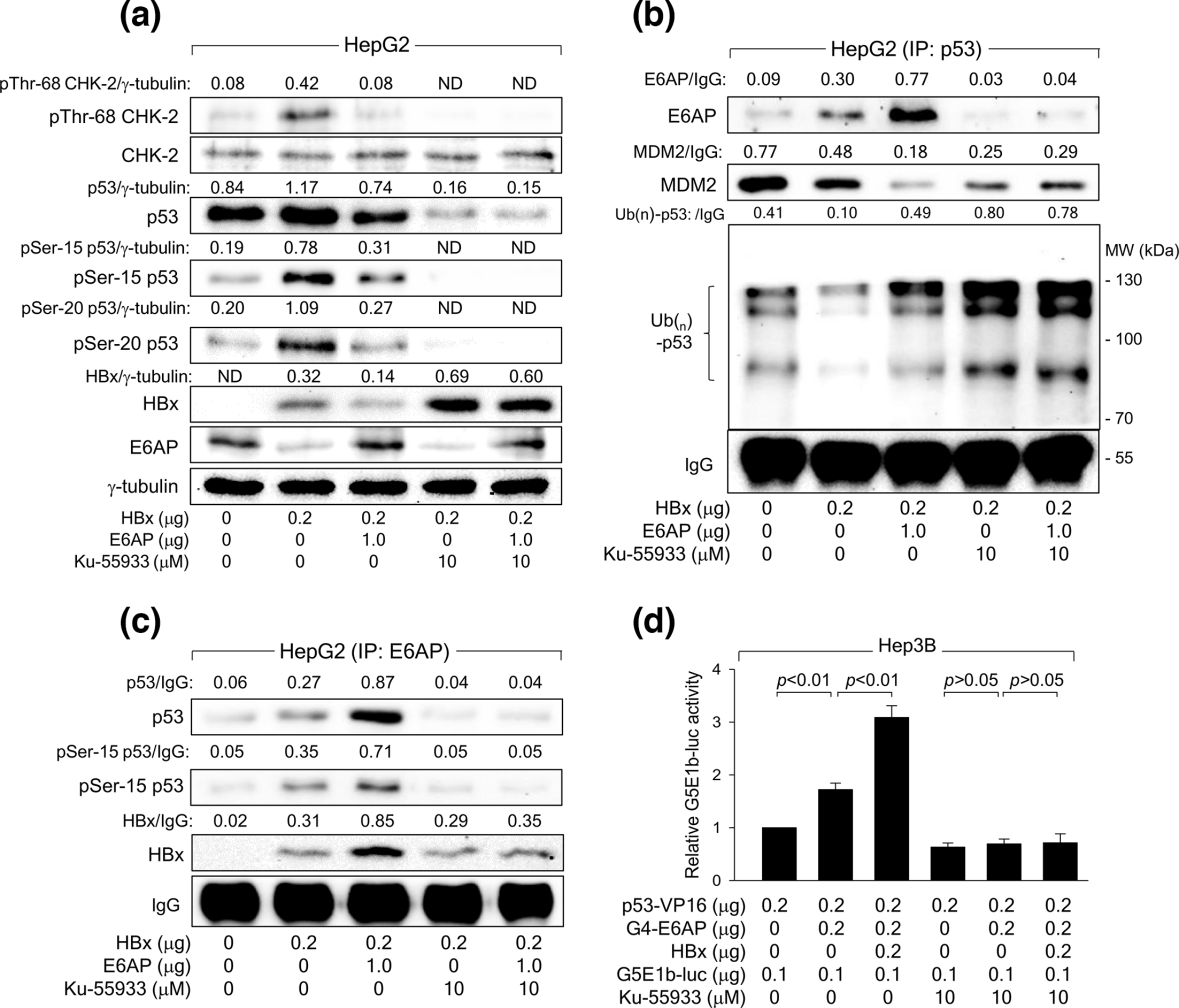
HBx-induced p53 phosphorylation is required for E6AP-mediated proteasomal degradation. (**a**) HepG2 cells transiently transfected with the designated plasmids for 47 h were treated with the indicated concentrations of the ATM inhibitor KU-55933 for 1 h before harvesting, followed by Western blotting. (**b,c**) Cell lysates prepared in (**a**) were immunoprecipitated with anti-p53 or anti-E6AP antibodies, followed by Western blotting. (**d**) For mammalian two-hybrid assays, Hep3B cells were transfected with pSG424-E6AP, pCMV p53-VP16 and G5E1b-luc, along with the indicated plasmids, for 47 h. Cells were either mock-treated or treated with 10 µM KU-55933 for 1 h before harvesting, followed by a luciferase assay (*n*=4).

To confirm that E6AP specifically drives ubiquitin-dependent proteasomal degradation of phosphorylated p53 in human hepatoma cells, we used the selective ATM inhibitor KU-55933, which blocks the ATM-Chk2 pathway [46]. Treatment with KU-55933 significantly reduced total p53 and phosphorylated forms of ATM, Chk2 and p53 (Fig. 4a). It also prevented E6AP from decreasing p53 levels in the presence of HBx. These results indicate that p53 phosphorylation is essential not only for HBx-induced upregulation of p53 levels but also for E6AP-mediated downregulation of p53 levels in the presence of HBx.

Next, we tested whether p53 phosphorylation is necessary for the interaction between E6AP and p53, as well as for E6AP-mediated p53 ubiquitination in the presence of HBx. Co-IP experiments using antibodies against p53 and E6AP showed that KU-55933 reduced the binding of E6AP to total and phosphorylated p53 in the presence of HBx to nearly undetectable levels (Fig. 4b, c). Additionally, ectopic E6AP expression did not increase its interaction with p53 under these conditions. In contrast, KU-55933 treatment significantly increased the interaction between MDM2 and p53 in the presence of HBx, resulting in increased levels of polyubiquitinated p53 in HepG2 cells (Fig. 4b). Mammalian two-hybrid assays also showed that KU-55933 nearly eliminated not only the interaction between p53 and E6AP but also the ability of HBx to enhance that interaction (Fig. 4d), supporting the co-IP findings (Fig. 4b, c). Based on these results, we conclude that p53 phosphorylation is essential for E6AP to function as an E3 ligase for p53 when HBx is present.

### HBx induces p53 ubiquitination either by promoting p53 phosphorylation or by facilitating E6AP’s binding to p53

**Fig. 5.**
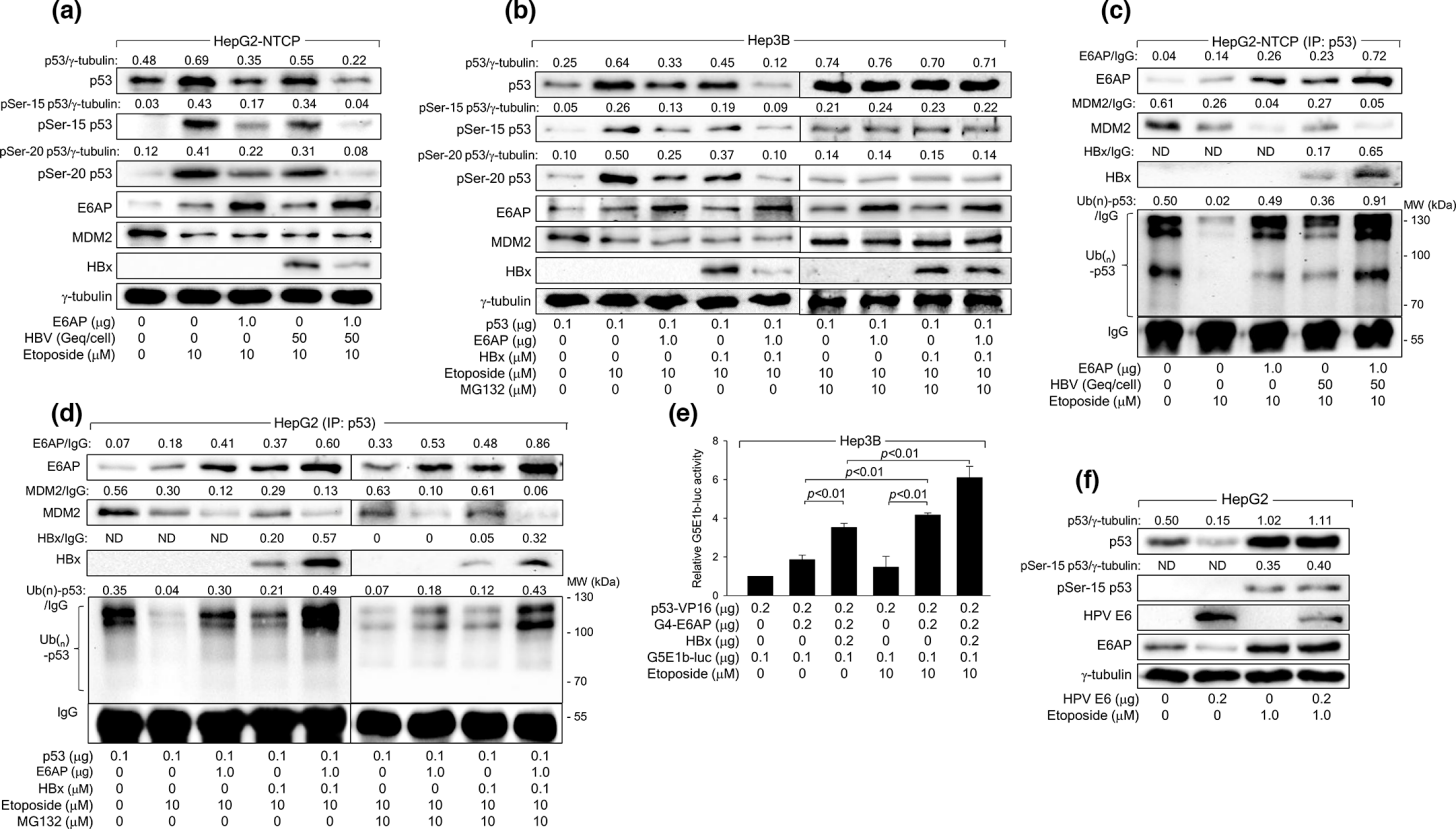
p53 phosphorylation is sufficient for E6AP to induce p53 ubiquitination in the presence of HBx. (**a, c**) HepG2-NTCP cells were either mock-infected or infected with WT HBV, as described in Fig. 1a. (**b, d**) HepG2 or Hep3B cells were transfected with an empty vector or an HBx expression plasmid, along with the indicated plasmids, for 48 h. (**f**) HepG2 cells were transfected with an empty vector or an HPV E6 expression plasmid for 48 h. (**a, b, f**) Protein levels were measured by Western blot analysis. (**c, d**) Total p53 was immunoprecipitated with an anti-p53 antibody and subjected to Western blotting with anti-E6AP, anti-MDM2, anti-HBx and anti-HA antibodies to detect E6AP, MDM2, HBx and HA-Ub-complexed p53, respectively. (**e**) For mammalian two-hybrid assays, Hep3B cells were transfected with pSG424-E6AP, pCMV p53-VP16 and G5E1b-luc, along with an empty vector or an HBx expression plasmid, for 48 h, followed by a luciferase assay (*n*=4). (a–f) Cells were treated with the designated concentrations of etoposide for 24 h and/or MG132 for 4 h before harvesting.

Our investigation aimed to determine whether phosphorylation of p53 alone is sufficient for E6AP to induce p53 ubiquitination. To address this, we used etoposide, a topoisomerase II inhibitor that induces dsDNA breaks and stabilizes p53 by activating the ATM-Chk2 pathway [47]. Etoposide induced p53 phosphorylation at Ser-15 and Ser-20 in HepG2-NTCP and Hep3B cells, increasing total p53 levels even in the absence of HBx (Fig. 5a, b). Additionally, neither HBV infection nor HBx expression further increased p53 levels in the presence of etoposide, likely because both HBx and etoposide stabilize p53 through the same ATM-Chk2 pathway. Notably, ectopic E6AP expression alone reduced total and phosphorylated p53 levels in etoposide-treated HepG2-NTCP and Hep3B cells without HBx. Therefore, the main role of HBx in E6AP-mediated proteasomal degradation of p53 appears to be inducing p53 phosphorylation. Interestingly, HBV infection and HBx expression lowered total and phosphorylated p53 levels in etoposide-treated cells regardless of ectopic E6AP expression (Fig. 5a, b), indicating that HBx also directly enhances E6AP-mediated proteasomal degradation of phosphorylated p53. Treatment with MG132 prevented E6AP from downregulating total and phosphorylated p53 in etoposide-treated cells, regardless of HBx presence (Fig. 5b), confirming that E6AP causes proteasomal degradation of phosphorylated p53 independently of HBx.

We next investigated the roles of HBx in E6AP-mediated p53 ubiquitination in both HBV infection and HBx overexpression systems. Etoposide treatment increased E6AP binding to p53, even in the absence of HBx, as shown by co-IP (Fig. 5c, d) and mammalian two-hybrid assays (Fig. 5e). However, this was offset by decreased MDM2 binding to p53, resulting in a significant reduction in p53 ubiquitination (Fig. 5c, d) and elevated p53 levels in etoposide-treated cells (Fig. 5a, b). Ectopic E6AP expression in the presence of etoposide strengthened its interaction with p53, regardless of HBx, while further decreasing MDM2–p53 binding, thereby increasing levels of polyubiquitinated p53 (Fig. 5c, d). Notably, HBx enhanced the interaction between E6AP and p53 in both co-IP (Fig. 5c, d) and mammalian two-hybrid assays (Fig. 5e), leading to higher levels of polyubiquitinated p53 in etoposide-treated cells (Fig. 5c, d). These effects were consistently observed under conditions in which MG132 maintained p53 and E6AP levels constant across samples (Fig. 5d). Based on these findings, we conclude that HBx promotes E6AP-mediated p53 ubiquitination by inducing p53 phosphorylation or by directly strengthening the interaction between E6AP and phosphorylated p53.

We examined whether HPV E6, which also promotes E6AP-mediated p53 ubiquitination [25,26], requires p53 phosphorylation to function as an E3 ligase. Unlike HBx, HPV E6 decreased p53 levels without inducing p53 phosphorylation (Fig. 5f). Additionally, HPV E6 did not alter p53 levels, whereas E6 levels were lowered in the presence of etoposide, which induces p53 phosphorylation. These findings suggest that HBx and E6 regulate p53 through distinct mechanisms, with HBx specifically controlling p53 degradation via phosphorylation-dependent pathways.

### HBx inhibits MDM2-mediated ubiquitination of p53 through phosphorylation

**Fig. 6.**
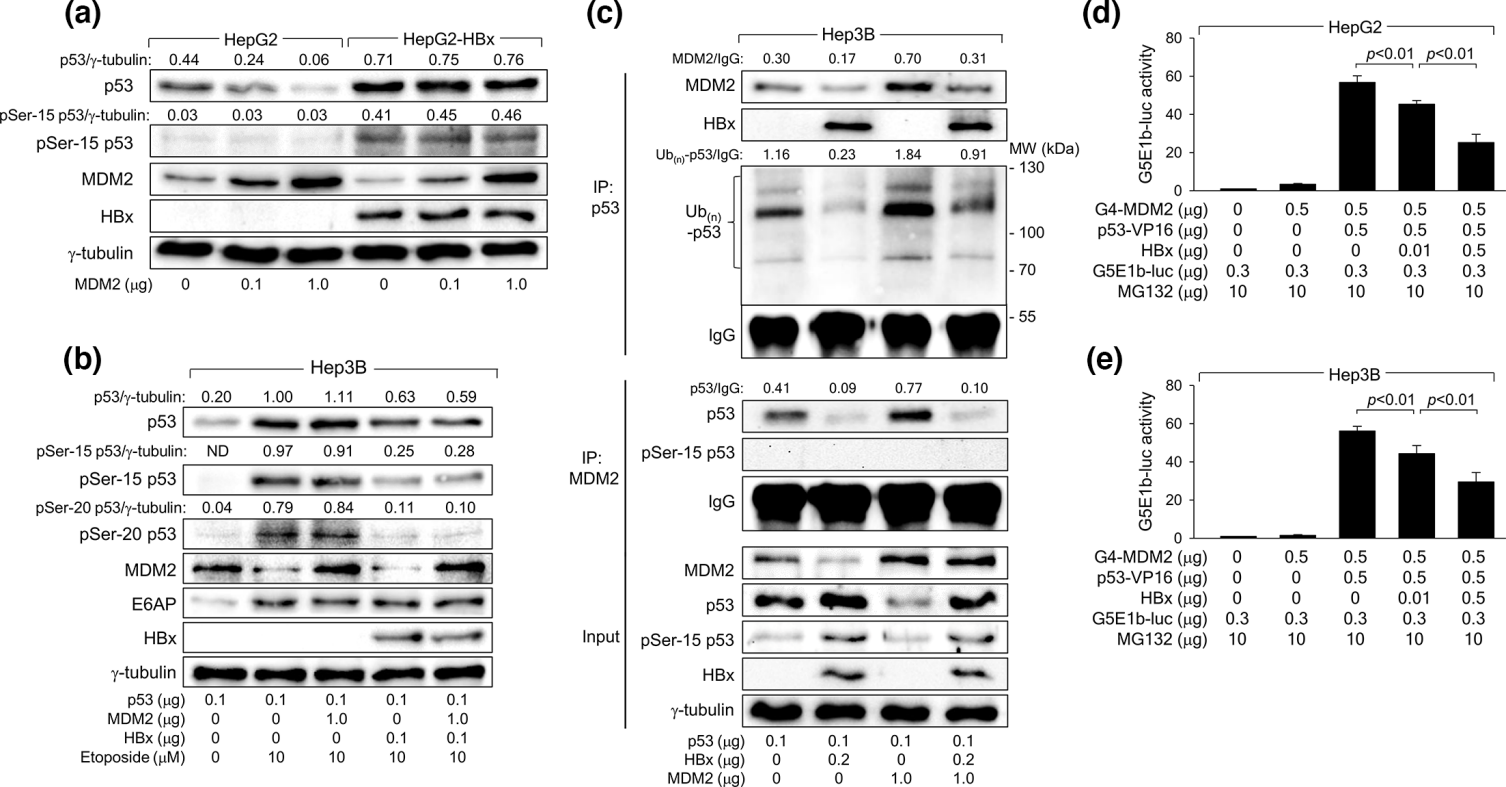
HBx inhibits MDM2-mediated proteasomal degradation of p53 through phosphorylation. (**a**) HepG2 and HdpG2-HBx cells were transiently transfected with increasing amounts of an MDM2 expression plasmid for 48 h, then analysed by Western blotting. (**b**) Hep3B cells were transfected with the indicated plasmids for 48 h. Cells were treated with the indicated concentrations of etoposide for 24 h before harvesting. (**c**) Hep3B cells were transfected with the indicated plasmids for 48 h. Total p53 in cell extracts was immunoprecipitated with an anti-p53 antibody and subjected to Western blotting with anti-MDM2, anti-HBx and anti-HA antibodies to detect MDM2, HBx and HA-Ub-complexed p53, respectively. The cell extracts were also subjected to co-IP with an anti-MDM2 antibody to measure levels of p53 and pSer-15 p53 complexed with MDM2. The input indicates the levels of the designated proteins in the cell lysates. (**d, e**) For mammalian two-hybrid assays, HepG2 and Hep3B cells were transfected with the Gal4 reporter (G5E1b-luc), pSG424-MDM2 and pCMV p53-VP16, along with either an empty vector or an HBx expression plasmid, for 48 h, followed by a luciferase assay. Cells were treated with 10 µM MG132 for 4 h before harvesting. The values show relative luciferase activity compared to the basal level (*n*=4).

The ability of HBx to prevent MDM2 from downregulating both total and phosphorylated p53, as shown in Fig. 3a, b, was more clearly demonstrated in HepG2 cells stably expressing HBx (Fig. 6a). The trend of decreased p53 levels upon MDM2 transfection in the presence of HBx (Fig. 3a, b) may result from incomplete HBx expression across the cell population. Additionally, ectopic MDM2 expression failed to reduce total and phosphorylated p53 levels, regardless of HBx presence, under conditions in which etoposide induces p53 phosphorylation (Fig. 6b). These findings suggest that MDM2 cannot mediate p53 degradation when p53 is phosphorylated by etoposide or HBx.

Co-IP experiments using anti-p53 and anti-MDM2 antibodies showed that MDM2 strongly interacts with p53 in the absence of HBx, resulting in p53 ubiquitination (Fig. 4c). However, these activities were significantly reduced in the presence of HBx. Ectopic MDM2 expression markedly increased the MDM2–p53 interaction, leading to p53 ubiquitination when HBx was absent but not when it was present. Data from a mammalian two-hybrid assay also indicated that HBx weakens the interaction between MDM2 and p53 in HepG2 and Hep3B cells (Fig. 6d, e). Furthermore, MDM2 did not interact with phosphorylated p53, regardless of HBx presence (Fig. 6c), as also demonstrated in Fig. 3c, d. These findings suggest that HBx inhibits MDM2-mediated p53 degradation by disrupting the MDM2–p53 interaction, primarily through p53 phosphorylation.

### Phosphorylation of p53 at Ser-15 is essential for E6AP-mediated degradation of p53

**Fig. 7.**
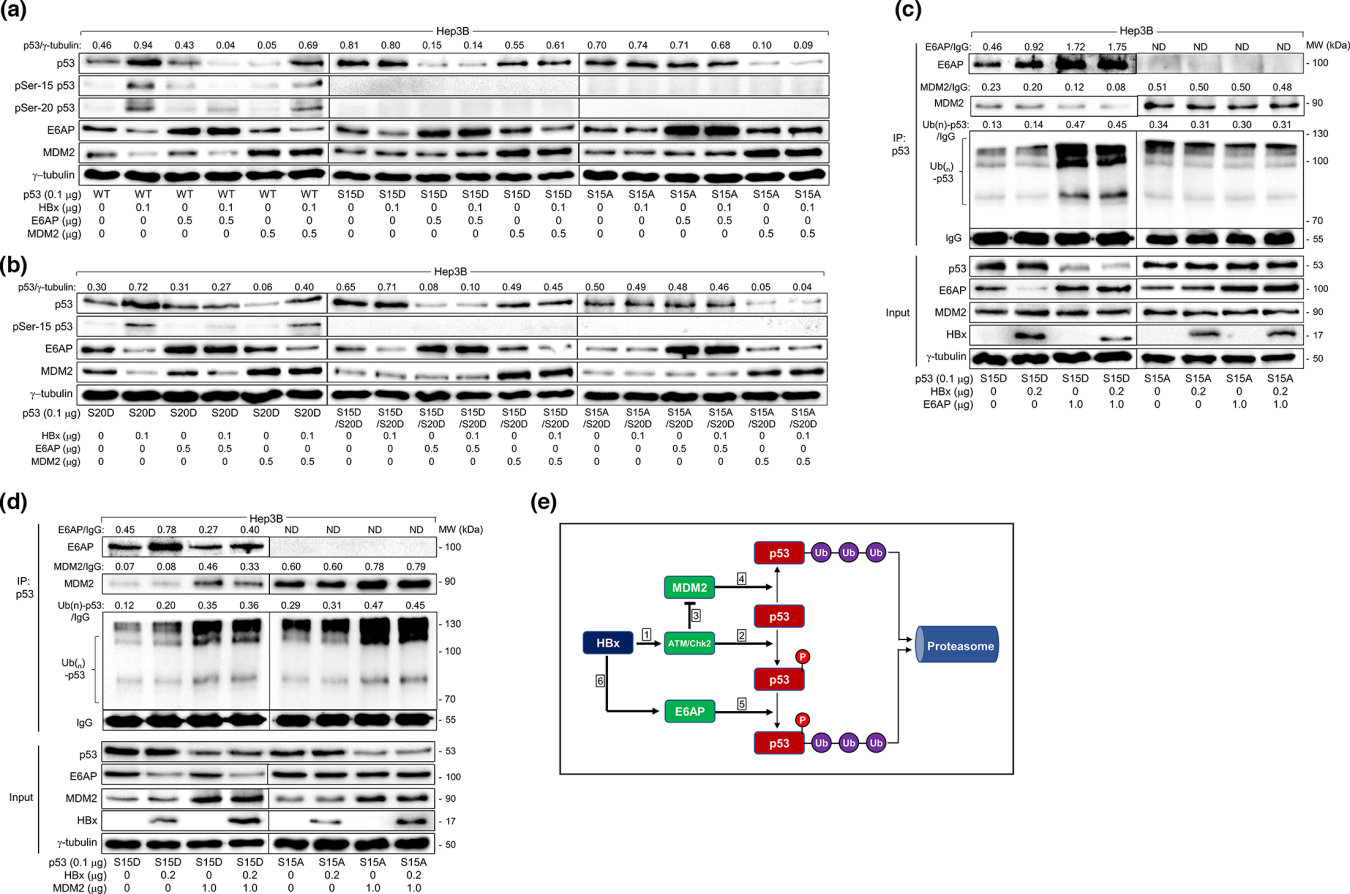
HBx-induced phosphorylation of p53 at Ser-15 is essential for E6AP-mediated degradation. (a–d) Hep3B cells were transfected with WT p53 or p53 mutants with substitutions at Ser-15 and/or Ser-20, along with the indicated plasmids, for 48 h. (**a, b**) Protein levels were assessed by Western blotting. (**c, d**) Cell lysates were immunoprecipitated with an anti-p53 antibody, and the immunoprecipitates were analysed by Western blotting. (**e**) Schematic diagram illustrating how HBx regulates the MDM2- and E6AP-mediated proteasomal degradation of p53. Each step is described in the Discussion section.

Results in Figs 3a, b and 5a, b show that both HBx expression and etoposide treatment promote phosphorylation of p53 at Ser-15 and Ser-20 in human hepatoma cells. To determine which phosphorylation site is critical for E6AP-mediated p53 degradation, we used several p53 mutants in which Ser-15 and/or Ser-20 were replaced with either a phosphomimetic amino acid (aspartate) or a non-phosphorylatable amino acid (alanine). HBx induced phosphorylation of p53 S20D at Ser-15, similar to that observed with WT p53 (Fig. 7a, b). However, HBx could not induce Ser-20 phosphorylation in p53 mutants with S15D or S15A substitutions, likely because Ser-20 phosphorylation depends on prior Ser-15 phosphorylation, as suggested by a sequential phosphorylation model [48,49]. These p53 mutants are therefore useful for studying the significance of phosphorylation at Ser-15 and Ser-20 in E6AP- and MDM2-mediated p53 degradation.

The susceptibility of each p53 mutant to E6AP- or MDM2-mediated downregulation was assessed with or without HBx. Ectopic E6AP expression reduced p53 S15D and p53 S15D/S20D, but not WT p53 or p53 mutants with S15A, S20D or S15A/S20D substitutions, in the absence of HBx (Fig. 7a, b). However, in the presence of HBx, ectopic E6AP still downregulated WT p53 and p53 S20D but not mutants with an S15A substitution. This indicates that E6AP can target p53 if it has either a phosphorylated Ser-15 or a phosphomimetic residue, but not a non-phosphorylatable residue at position 15. Thus, phosphorylation at Ser-15 on p53 is both necessary and sufficient for E6AP-mediated degradation. Ectopic MDM2 effectively downregulated p53 S15A and p53 S15A/S20D, regardless of the presence of HBx (Fig. 7a, b). It also decreased p53 S20D in the absence of HBx but not when HBx was present (Fig. 7b), as shown with WT p53 (Fig. 7a). Notably, MDM2 could downregulate p53 S15D and p53 S15D/S20D regardless of HBx, but with a much lower impact compared to p53 mutants with an S15A substitution (Fig. 7a, b). These findings suggest that phosphorylation at Ser-15 alone does not fully prevent degradation but provides partial resistance to MDM2-mediated degradation, as previously shown [48,49]. HBx did not influence E6AP-mediated downregulation of p53 mutants with either a phosphomimetic or a non-phosphorylatable residue at position 15 (Fig. 7a, b). Similarly, HBx did not affect MDM2’s ability to downregulate p53 mutants, except for p53 S20D, where Ser-15 is phosphorylated by HBx. Based on these results, we conclude that phosphorylation at Ser-15 is necessary for HBx to inhibit the ability of MDM2 to suppress p53 and to promote E6AP-mediated p53 degradation.

Next, our study aimed to determine whether Ser-15 phosphorylation affects E6AP-mediated p53 ubiquitination in the presence or absence of HBx. p53 S15D, but not p53 S15A, binds E6AP with high affinity (Fig. 7c), indicating that phosphorylation at Ser-15 is essential for E6AP binding. Ectopic E6AP expression further increased binding to p53 S15D, while slightly decreasing the interaction between MDM2 and p53 S15D, leading to a significant increase in p53 ubiquitination. However, E6AP had minimal effect on E6AP or MDM2 binding to p53 S15A and did not affect ubiquitination of p53 S15A (Fig. 7c). The effects of HBx on the interaction between E6AP and p53 S15D, as well as on ubiquitination, were limited or undetectable (Fig. 7c, d), compared to those with WT p53 (Fig. 2c). Similarly, HBx had minimal effects on E6AP binding to p53 S15A and on its ubiquitination (Fig. 7c). These findings indicate that phosphorylation at Ser-15 is crucial for E6AP binding and subsequent p53 ubiquitination in the presence of HBx.

Finally, we examined whether Ser-15 phosphorylation affects MDM2-mediated p53 ubiquitination in the presence or absence of HBx. p53 S15D clearly interacted with MDM2, but at much lower levels than p53 S15A (Fig. 7c, d), indicating that phosphorylation at Ser-15 alone weakens, but does not prevent, the interaction between MDM2 and p53. Overexpressing MDM2 increased its binding to both p53 S15D and p53 S15A, causing a significant increase in their ubiquitination, although the increase was much more noticeable with p53 S15A (Fig. 7d). The effects of HBx on the interaction between MDM2 and p53 S15D and on its ubiquitination were minimal or undetectable (Fig. 7d), likely because of HBx’s limited ability to bind MDM2 (Fig. 7c, d). Similarly, the effects of HBx on MDM2 binding to p53 S15A and on ubiquitination were marginal (Fig. 7c, d), unlike its effects on WT p53 (Fig. 6c). This is probably because p53 S15A can still interact effectively with MDM2 even in the presence of HBx (Fig. 7c, d). These results suggest that phosphorylation at Ser-15 is essential for HBx to inhibit MDM2-mediated degradation of p53.

## Discussion

The tumour suppressor protein p53 is essential for protecting the host against viral infections by blocking viral replication and preventing cell transformation [50,51]. Viruses have evolved strategies to inactivate p53, such as degrading the protein or impairing its function, thereby evading antiviral defenses and replicating more effectively. For instance, SV40 large T antigen binds p53, forming a stable complex that blocks its tumour-suppressing functions [18]. Similarly, HBx interacts with p53 to interfere with several key p53 functions [19,21]. In contrast, the adenovirus E1B 55 kDa protein binds both p53 and E4orf6, recruiting a Cullin-based E3 ubiquitin ligase that facilitates the ubiquitin-dependent degradation of p53 [18]. Likewise, the HPV E6 protein and hepatitis C virus core protein recruit E6AP to promote ubiquitination and proteasomal degradation of p53 [15,25, 26]. Our current study shows that HBx also triggers E6AP-mediated ubiquitination and proteasomal degradation of p53. Overall, p53 degradation by the human proteasome appears to be a common mechanism by which some human tumour viruses evade p53’s antiviral actions.

Co-IP assays consistently show that HBx interacts with E6AP and p53 to form a trimeric complex, leading to p53 ubiquitination (Fig. 2c–f). Therefore, our findings support the established mechanism by which HPV E6 promotes E6AP-mediated ubiquitination and proteasomal degradation of p53 [25,26]. However, the detailed mechanisms of HPV E6 and HBx appear to differ. In a trimeric complex, HPV E6 alters the conformation of E6AP, thereby activating it to induce p53 ubiquitination and proteasomal degradation [28,29]. In contrast, HBx primarily contributes to E6AP-mediated p53 degradation by inducing its phosphorylation via activation of the ATM-Chk2 pathway (Fig. 3c, d; Fig. 7e, steps 1, 2 and 5). Several lines of evidence suggest that p53 phosphorylation is both necessary and sufficient for E6AP-mediated p53 ubiquitination in the presence of HBx. First, treatment with the ATM inhibitor KU-55933 prevented E6AP from binding to p53 and promoting its ubiquitination in the presence of HBx (Fig. 4b–d). Second, treatment with etoposide induced p53 phosphorylation via activation of the ATM-Chk2 pathway, thereby enabling E6AP to promote p53 ubiquitination even in the absence of HBx (Fig. 5c–e), as previously shown [30]. Accordingly, the ability of HBx to induce ubiquitination of p53 was largely diminished in the presence of etoposide (Fig. 5c, d). Third, E6AP induced ubiquitination of p53 mutants with an aspartic acid at Ser-15 in the absence of HBx (Fig. 7c), as previously shown [30]. As a result, HBx failed to induce ubiquitination of p53 S15D (Fig. 7c). In contrast, ectopic E6AP expression did not induce noticeable changes in protein levels of p53 S20D and p53 S15A/S20D in the absence of HBx, as observed with WT p53 (Fig. 7a, b), suggesting that p53 phosphorylation at Ser-20 alone is not sufficient to confer susceptibility to E6AP-mediated degradation. As p53 S15D/S20D was more susceptible than p53 S15D to E6AP-mediated degradation in the absence of HBx, it is likely that phosphorylation of p53 at Ser-20 augments the susceptibility of p53 phosphorylated at Ser-15. Taken together, the capacity of HBx to induce p53 ubiquitination appears largely to depend on its ability to phosphorylate p53, particularly at Ser-15. Therefore, HBx, unlike HPV E6, does not have to directly interact with E6AP to enhance E6AP’s enzymatic activity for p53 ubiquitination.

The ability of HBx to form a trimeric complex with E6AP and p53 may represent an additional mechanism that enhances E6AP-mediated ubiquitination of phosphorylated p53. HBx promotes E6AP-mediated p53 ubiquitination by strengthening the interaction between phosphorylated p53 and E6AP (Fig. 5c–e; Fig. 7e, steps 5 and 6). However, the effects of HBx on the interaction between E6AP and p53 S15D, as well as on its ubiquitination, were minimal or undetectable (Fig. 7c, d), likely because p53 S15D, unlike WT p53, can optimally interact with E6AP without the help of HBx (Fig. 7c, d). Unlike HBx, HPV E6 could not induce phosphorylation of p53 (Fig. 5f). It remains unknown whether HPV E6, in a trimeric complex, alters the conformation of E6AP and/or unphosphorylated p53 through physical interactions that can meet the p53 phosphorylation requirement. Additionally, HPV E6 could not promote the degradation of phosphorylated p53 in the presence of etoposide, which is attributable in part to its low levels (Fig. 5f). Further studies are necessary to understand the structural differences in their interactions with p53 and E6AP.

Distinct mechanisms by which E6AP and MDM2 target p53 further underscore the distinct roles of HBx in p53 regulation. The levels of E6AP- and MDM2-bound p53 were inversely correlated across all experimental conditions, suggesting that E6AP and MDM2 exert antagonistic effects on p53 ubiquitination. However, several lines of evidence exclude the possibility that E6AP and MDM2 directly compete for binding to p53 to act as a major E3 ligase. First, ectopic E6AP expression decreased the interaction between MDM2 and p53 in the presence of HBx but not in its absence, whereas ectopic MDM2 expression decreased the interaction between E6AP and p53 in the absence of HBx but not in its presence (Fig. 2c, d), suggesting distinct roles of HBx in E6AP- and MDM2-mediated p53 regulation. Second, MDM2, but not E6AP, effectively bound to p53 in the presence of KU-55933, which prevented HBx from inducing p53 phosphorylation (Fig. 4b). Third, E6AP, but not MDM2, preferentially bound to p53 in the presence of etoposide, which induces p53 phosphorylation without HBx (Fig. 5c, d). Fourth, E6AP, but not MDM2, could bind phosphorylated p53 (Fig. 3c, d) and p53 mutants with an S15D substitution (Fig. 7c, d), thereby inducing their ubiquitination. In contrast, MDM2, but not E6AP, could induce ubiquitination of p53 mutants with an S15A substitution, regardless of the presence of HBx (Fig. 7c, d). Therefore, MDM2 is likely specialized for unphosphorylated p53, whereas E6AP targets either phosphorylated p53 or p53 bound to a viral oncoprotein such as HPV E6. This dynamic interplay between E6AP and MDM2 likely involves HBx acting as a molecular switch that selectively enhances or inhibits each E3 ligase’s ability to target p53, depending on the cellular context.

Based on this study’s findings, the roles of HBx can be summarized as follows: first, HBx induces p53 phosphorylation and blocks MDM2-mediated p53 ubiquitination, thereby increasing p53 levels [16,24] (Fig. 7e, steps 1–4). Second, HBx-induced p53 phosphorylation promotes the interaction between E6AP and p53, leading to E6AP-mediated ubiquitination of p53 (Fig. 7e, steps 1, 2, 5 and 6). The reason HBx has dual effects on p53 stability remains unclear. If p53 phosphorylation via the ATM-Chk2 pathway is part of the host’s antiviral response to HBV, then E6AP-mediated degradation of phosphorylated p53 could be a viral tactic to bypass the host’s p53-driven defences. Alternatively, this dual regulation might ensure that p53 levels temporarily rise to activate cellular stress responses before being targeted for degradation, causing fluctuations in both HBV and p53 during infection. Regardless of the mechanism, these findings reveal the complex role of HBx in precisely controlling p53 levels, thereby creating an environment favourable to persistent HBV infection.

## Abbreviations

ATM: ataxia telangiectasia mutated
Chk2: checkpoint kinase 2
CHX: cycloheximide
co-IP: co-immunoprecipitation
DMEM: Dulbecco’s modified Eagle medium
E6AP: E6-associated protein
HA: haemagglutinin
HBV: hepatitis B virus
HBx: HBV X protein
HCC: hepatocellular carcinoma
HECT: homologous to the E6-AP Carboxyl Terminus
HPV: human papillomavirus
NTCP: Na^+^-taurocholate cotransporting polypeptide
PEG: polyethylene glycol
pSer-15 p53: p53 phosphorylated at Ser-15
shRNA: small hairpin RNA
β-Gal: β-galactosidase
